# *MCPH1* and DNA Damage Response Pathway Genes as Candidate Susceptibility Loci in Hereditary Breast Cancer

**DOI:** 10.3390/diagnostics16182967

**Published:** 2026-09-14

**Authors:** Altuğ Koç, Veysel Atasoy, Özge Özer Kaya, Selcan Keşan, Merve Saka, Mehmet Berkay Akcan, Taha Reşid Özdemir, Kadri Murat Erdoğan, Özlem Boz, Berk Özyılmaz, Olçun Ümit Ünal, Umut Çakıroğlu, Seval Akay, Mehmet Erdinç Esenkaya

**Affiliations:** 1 Genetic Diagnosis and Evaluation Center, Izmir City Hospital, University of Health Sciences, Izmir Faculty of Medicine, Izmir 35540, Turkey; dratasoyveysel@gmail.com (V.A.); dr.tahaoz@gmail.com (T.R.Ö.); drberk@gmail.com (B.Ö.); 2 Medical Oncology Department, Izmir City Hospital, University of Health Sciences, Izmir Faculty of Medicine, Izmir 35540, Turkey; drolcun@hotmail.com (O.Ü.Ü.); drsevalakay@hotmail.com (S.A.);

**Keywords:** breast cancer, *MCPH1*, *BRIT1*, DNA damage response, hereditary breast and ovarian cancer, *ATRIP*, *RAD54L*, *MDC1*, germline variants, next-generation sequencing

## Abstract

**Background/Objectives:** Standard hereditary breast cancer gene panels fail to identify causative variants in some patients meeting hereditary breast and ovarian cancer (HBOC) criteria. This exploratory, hypothesis-generating study characterised germline variants in *MCPH1*, functionally related DNA damage response genes, and selected low-evidence candidate genes in breast cancer patients with uninformative standard panel results. **Methods:** A total of 185 patients meeting NCCN/ACMG criteria, with normal 42-gene panel and *BRCA1/2*/*PALB2* MLPA results, underwent second-tier targeted sequencing. Group 1 comprised *MCPH1* and related genes (*TP53BP1*, *MDC1*, *TOPBP1*, *UBE2N*, *RAD52*); Group 2 consisted of Genomics England PanelApp candidates (*ATRIP*, *ESR1*, *PPM1D*, *RAD54L*, *RRAS2*, *XRCC2*). No ethnically matched control cohort was available. Variants were classified per ACMG 2015 guidelines and confirmed by Sanger sequencing. **Results:** Rare variants meeting reporting criteria were identified in 20/185 patients (10.8%): one likely pathogenic (LP) *MCPH1* frameshift deletion (c.1869_1870del) in a luminal B invasive ductal carcinoma patient, nineteen variants of uncertain significance (VUS), and one unconfirmed copy number variant. *ATRIP* was the most frequently implicated Group 2 gene (4 patients), including a homozygous variant; *RAD54L* variants were identified in three patients, including the only male breast cancer case. **Conclusions:** These descriptive findings identify *MCPH1*, *ATRIP*, *MDC1*, *TOPBP1*, and *RAD54L* as candidates warranting evaluation in case–control studies with ancestry-matched cohorts, functional validation, and family segregation analysis before any consideration for panel inclusion.

## 1. Introduction

Hereditary breast and ovarian cancer (HBOC) is a significant clinical concern, as breast cancer is the most common cancer among women worldwide and remains one of the leading causes of cancer-related death [1]. Roughly one in five familial HBOC cases are attributable to pathogenic variants in two well-established genes, *BRCA1* and *BRCA2* [2]. Beyond these, research over recent decades has identified additional genes associated with elevated cancer risk, which can be broadly grouped into three categories: high-penetrance genes such as *BRCA1*, *BRCA2*, *PALB2*, and *TP53*; moderately penetrant genes like *CHEK2* and *ATM*; and common low-risk variants that, when combined, form a polygenic risk score [2]. Breast cancer, in which the complex interaction of environmental, hormonal, and genetic factors plays a role in its etiology, is a highly heterogeneous disease both clinically and molecularly. Approximately 10–15% of all breast cancer cases arise from germline pathogenic variants in specific genes and are defined as “hereditary breast cancer” [3]. Elucidating hereditary breast cancer syndromes is of critical importance both for the personalised treatment of patients and for the identification and follow-up of at-risk family members [2]. In daily practice, many different gene panels are used in the investigation of familial breast cancer, and the majority of these do not overlap beyond the well-known high-penetrance genes.

The composition of clinical gene panels is increasingly guided by standardised evidence frameworks rather than by biological plausibility alone. The Clinical Genome Resource (ClinGen) Gene–Disease Validity framework appraises gene–disease relationships on the basis of genetic and experimental evidence, classifying them from Definitive through Limited to No Known Disease Relationship [4]. Genes with evidence classified below Moderate are generally considered to have limited clinical validity and to warrant additional evidence before consideration for routine clinical implementation, as reporting their variants may otherwise generate clinically unactionable findings. The candidate genes examined here fall largely into this lower-evidence category, being supported mainly by mechanistic data or anecdotal case reports, which explains their absence from routine clinical panels. The present study was therefore designed to generate the case-level human genetic data that are currently lacking, rather than to advocate immediate clinical implementation of these genes.

Population-specific genetic background also influences diagnostic yield and variant interpretability. In Turkish high-risk or *BRCA1/2*-negative cohorts, *ATM* is consistently among the most frequently implicated genes beyond *BRCA1/2* [5,6], while studies conducted in the Aegean region—the source of the present cohort—have described candidate *BRCA1* founder alleles and locally divergent allele frequencies [7]. Consistent with the high proportion of previously unreported *BRCA1/2* variants in Turkish patients [8], this population may harbour rare or population-specific alleles that remain underrepresented in global databases such as gnomAD.

The search for genetic alterations involved in the etiology of breast cancer is ongoing. Among these, pathogenic variants in the *MCPH1* gene (Microcephalin 1, also known as *BRIT1*) are of particular interest. The *MCPH1* gene encodes a multifunctional protein essential for brain development, genomic, and tumour suppression, regulating neurogenesis, DNA damage repair, cell cycle progression, and mitochondrial metabolism. Its N-terminal BRCT domain is essential for neuroprogenitor cell cycle regulation and chromatin remodelling, preventing premature differentiation and ensuring correct cerebral cortex size. *MCPH1* acts as a tumour suppressor by orchestrating the DNA damage response through recruitment of repair proteins (*BRCA1*, CHK1, RAD51), and links to mitochondrial metabolism via VDAC1/GRP75 and ATF4/PCK2 pathways. Mutations in *MCPH1* are associated with neurological deficits (e.g., primary microcephaly), infertility, and various cancers, underlining its broad medical importance [9,10,11,12].

**Figure 1:**
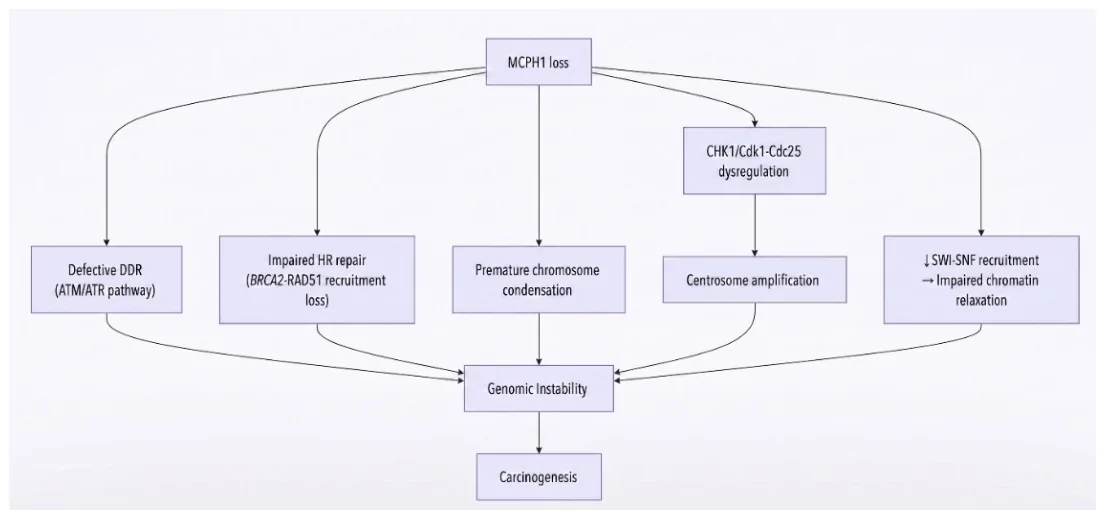
Diagram illustrating how MCPH1 deficiency may lead to carcinogenesis via defects in DNA damage response, homologous recombination, centrosome regulation, and chromosome condensation. SWI/SNF: SWItch/Sucrose Non-Fermentable.

Encountering a breast cancer case carrying a pathogenic *MCPH1* variant at our centre (to be reported separately) prompted the present study. As human case-level data linking these genes to breast cancer are scarce, candidate selection could not rely on established gene–disease associations and was instead guided by functional relatedness to *MCPH1*; genes acting in the same DNA damage response and homologous recombination pathways (*TP53BP1*, *MDC1*, *TOPBP1*, *UBE2N*, *RAD52*) were included as Group 1 (Figure 1). This reflects a hypothesis-generating rather than a hypothesis-testing design. Group 2 comprised low-evidence candidates not included in routine panels (*ATRIP*, *ESR1*, *PPM1D*, *RAD54L*, *RRAS2*, *XRCC2*), derived from the Genomics England PanelApp Familial Breast Cancer list.

In the present study we applied a two-stage (second-tier) testing strategy: patients meeting NCCN/ACMG criteria first underwent routine 42-gene hereditary cancer panel and *BRCA1/2*/*PALB2* MLPA testing, and only those with uninformative results proceeded to analysis of the Group 1 and Group 2 candidate genes. This design focuses investigation on genetically unresolved cases while minimising detection of variants in genes whose etiological contribution is already established. The aim was to describe the spectrum and frequency of rare germline variants in these candidates within a regionally defined cohort and to evaluate the findings in the light of the literature. Given the descriptive design and the absence of an ethnically matched control cohort, the study was not intended to establish gene–disease associations; the findings should be interpreted as preliminary observations intended to inform future functional, segregation, and case–control studies.

## 2. Materials and Methods

### 2.1. Study Design and Patients

This study enrolled 514 patients with suspected hereditary breast cancer—defined according to National Comprehensive Cancer Network (NCCN) and American College of Medical Genetics and Genomics (ACMG) clinical criteria as individuals presenting with early-onset diagnosis, a family history of multiple cancers, or bilateral breast tumours—along with their at-risk relatives, between February 2026 and May 2026. A hereditary cancer panel was performed in all 514 patients; of these, 185 patients with a confirmed diagnosis of breast cancer met the study inclusion criteria and were included in the molecular analysis. The majority of the patient population originated from the Aegean Region of Turkey. Most patients were female; only two male cases with breast cancer were included. The mean age at referral was 53.08 years, and the mean age at diagnosis was 51.66 years.

A two-stage (second-tier) testing strategy was applied. In the first stage, all patients underwent routine diagnostic evaluation with a 42-gene hereditary cancer panel followed, where negative, by *BRCA1*, *BRCA2*, and *PALB2* MLPA analysis. In the second stage, only those patients whose first-stage evaluation remained uninformative proceeded to targeted sequencing of the Group 1 and Group 2 candidate genes. This selective design concentrates the analysis on genetically unresolved cases and minimises the detection of variants in genes whose etiological contribution to hereditary breast cancer is already established.

This study was designed as a descriptive, hypothesis-generating investigation. An ethnically matched, cancer-free control cohort was not available at our centre, and consequently no case–control burden analysis, gene-based enrichment testing, or estimation of association measures (odds ratios, confidence intervals, or *p*-values) was undertaken. Variant frequencies observed in the study cohort were instead compared descriptively against three reference datasets—gnomAD, the Turkish national variant database, and our in-house variant database—which provide a frequency context for the observed alleles but do not permit formal statistical inference regarding disease association. This constraint is explicitly acknowledged in the Limitations section.

### 2.2. DNA Extraction and Routine Hereditary Cancer Panel

Genomic DNA was extracted from peripheral blood samples collected from enrolled patients using a standardised protocol (Roche MagNA Pure 96 DNA and Viral NA Small Volume Kit, Mannheim, Germany). Extracted DNA samples were analysed using a custom-designed hereditary cancer panel comprising the following genes: *APC*, *ATM*, *AXIN2*, *BAP1*, *BARD1*, *BLM*, *BMPR1A*, *BRCA1*, *BRCA2*, *BRIP1*, *CDH1*, *CDKN2A*, *CHEK2*, *EPCAM*, *FH*, *FLCN*, *GALNT12*, *HOXB13*, *MEN1*, *MLH1*, *MSH2*, *MSH3*, *MSH6*, *MUTYH*, *NBN*, *NF1*, *NF2*, *NTHL1*, *PALB2*, *PMS2*, *POLD1*, *PTEN*, *RAD51C*, *RAD51D*, *RB1*, *RET*, *SMAD4*, *STK11*, *TP53*, *TSC1*, *TSC2*, and *VHL* (Table S1). All coding regions and exon–intron boundaries (±10 bp) of the listed genes were sequenced using the MGI DNBSEQ-G400 platform (MGI Tech, Shenzhen, China) and custom design sequencing test kit. Patients with a negative panel result subsequently underwent *BRCA1*, *BRCA2*, and *PALB2* multiplex ligation-dependent probe amplification (MLPA) analysis (MRC Holland, Amsterdam, The Netherlands).

### 2.3. Second-Tier Candidate Gene Analysis

Patients whose routine genetic evaluation remained uninformative underwent second-tier targeted sequencing of two additional gene groups, using the same laboratory infrastructure:

The first group comprised *MCPH1* and its functionally related genes, identified using the GenesLikeMe online tool (https://glm.genecards.org) based on similarity relationships across protein domains, super pathways, phenotypes, compounds, diseases, and gene ontologies. Genes yielding the highest overall similarity scores (ranging from 3.04 to 2.04) were: *TP53* (3.04), *BRCA1* (2.75), *NBN* (2.44), *TP53BP1* (2.39), *PARP1* (2.31), *MDC1* (2.19), *TOPBP1* (2.17), *UBE2N* (2.13), and *RAD52* (2.04). Among these, genes not currently included in standard hereditary cancer panels—namely *MCPH1*, *TP53BP1*, *MDC1*, *TOPBP1*, *UBE2N*, and *RAD52*—were selected for inclusion in this study.

The second group consisted of candidate genes with low-evidence associations with breast cancer that are not routinely included in standard hereditary cancer panels in clinical practice. These genes were selected from the Genomics England PanelApp Familial Breast Cancer gene list (Version 1.28; https://panelapp.genomicsengland.co.uk/panels/158/, accessed on 1 February 2026) and included: *ATRIP*, *ESR1*, *PPM1D*, *RAD54L*, *RRAS2*, and *XRCC2*.

### 2.4. Bioinformatic Analysis and Variant Classification

Raw sequencing data generated by NGS analysis were aligned to the GRCh38 (hg38) reference genome, and variant calling was performed using the Genomize SEQ Platform v9.6.0 (Istanbul, Turkey). The Integrative Genomics Viewer (IGV v2.19.7; Broad Institute, Cambridge, MA, USA) was used to verify data quality and read alignments. The following quality thresholds were applied during variant filtering: Low-confidence variants and regions with a read depth below 20× were excluded from analysis. The mean read depth across the panel was 150×, and target region coverage at 50× read depth exceeded 99%. Intronic variants outside coding and splice regions, synonymous variants, and strand bias artifacts were filtered out.

The clinical significance of variants passing quality filters was assessed using internationally recognised databases and tools, including ClinVar, HGMD, gnomAD, VarSome, Franklin, and an in-house variant database. Final variant classification was performed in accordance with the 2015 American College of Medical Genetics and Genomics and Association for Molecular Pathology (ACMG/AMP) guidelines [13], categorising variants into five tiers: Pathogenic (P), Likely Pathogenic (LP), Variant of Uncertain Significance (VUS), Likely Benign (LB), and Benign (B).

### 2.5. Orthogonal Confirmation

In line with the clinical next-generation sequencing testing recommendations of the College of American Pathologists (CAP) [14] and the American College of Medical Genetics and Genomics (ACMG) [15], both of which require confirmation of clinically reported NGS findings by an independent methodology, all 20 sequence variants (single-nucleotide variants and small indels) reported in this study were subsequently validated by Sanger sequencing. Complete concordance (100%) was observed between the NGS and Sanger results for these 20 variants. As copy number variants cannot be confirmed by Sanger sequencing, the copy number variant identified in CASE 20 was not validated by an independent method (see Section 2.6).

### 2.6. Technical Limitations of Variant Detection

Copy number variation was assessed from the targeted panel sequencing data using the read-depth-based algorithm implemented in the Genomize SEQ Platform. Detection of copy number alterations from targeted panel data is inherently constrained by probe density and coverage uniformity, and single-exon events in particular require orthogonal confirmation before clinical interpretation. The copy number finding reported in this study should therefore be regarded as provisional pending confirmation by an independent method such as MLPA or array-based analysis.

The sequencing strategy employed here targeted coding regions and exon–intron boundaries (±10 bp) only. Consequently, large structural rearrangements such as balanced translocations and inversions, as well as deep intronic variants that may have functional significance, were not analysed, and pathogenic variants located outside the sequenced intervals may have been missed. The present study therefore does not claim comprehensive coverage of all possible classes of germline alteration in the analysed genes.

### 2.7. Use of Artificial Intelligence Tools

In our study, AI-assisted technologies (Claude Sonnet 4.6, Anthropic, San Francisco, CA, USA) were used only to improve the readability and language of the manuscript text and to assist in the preparation of Figure 1. AI-assisted technologies were not involved in the study design, data analysis, data interpretation, authorship, or editorial decision-making processes. The authors have reviewed and edited all output and take full responsibility for the content of this publication.

## 3. Results

### 3.1. Patient Characteristics and Testing Workflow

A total of 185 patients with a breast cancer diagnosis who met NCCN and ACMG clinical criteria for hereditary breast cancer suspicion were enrolled in the second-tier analysis. All 185 patients had previously received uninformative results from the first-tier evaluation, comprising the routine 42-gene hereditary cancer panel and *BRCA1*/*BRCA2*/*PALB2* MLPA testing; patients in whom a pathogenic or likely pathogenic variant had been identified at the first tier were not carried forward, so that the second-tier analysis was restricted to genetically unresolved cases. The cohort was predominantly female (183/185; 98.9%), with only two male breast cancer cases.

Additional baseline characteristics for the full cohort were retrospectively extracted from clinical records. The mean age at diagnosis was 51.2 ± 11.1 years (median 50, interquartile range 43–60; range 26–79); 87 patients (47.0%) were diagnosed before age 50, and 98 (53.0%) at or after age 50. A family history of cancer of any type was present in 142 patients (76.8%), including a family history of breast cancer specifically in 77 patients (41.6%), with an affected first-degree relative in 22 patients (11.9%). Bilateral breast cancer was confirmed in 5 patients (2.7%); as unilaterality was not consistently documented in the clinical notes, this figure should be regarded as a minimum estimate. Molecular subtype could be assigned, based on ER/PR/HER2 and Ki-67 status recorded in the clinical notes, in 94 of 185 patients (50.8%); subtype data were unavailable for the remaining patients, chiefly owing to missing Ki-67 values, and are therefore not reported at the cohort level.

### 3.2. Overall Variant Detection Rate

Rare germline variants meeting the reporting criteria were identified in 20 of 185 patients (10.8%). A total of 21 distinct variants were detected across both gene groups, as one patient (CASE 3) carried concurrent variants in two genes (*MCPH1* and *ESR1*). Among all detected variants, one was classified as LP, 19 as VUS, and one as a copy number variant (CNV) of uncertain significance. No pathogenic (Class 5) variants were identified in the study genes. No variants of any classification were detected in *UBE2N*, *RRAS2*, or *XRCC2*. This 10.8% figure denotes the overall variant detection rate across all classifications and should not be equated with a clinical diagnostic yield: only the MCPH1 c.1869_1870del frameshift deletion met criteria for a clinically actionable (likely pathogenic) classification, corresponding to a true diagnostic yield of 1/185 patients (0.5%).

Of the 21 variants, 20 were sequence variants (single-nucleotide variants or small indels), all of which were confirmed by Sanger sequencing with complete concordance between the two methods. The remaining finding, a copy number variant (CASE 20), was not confirmed by an independent method and is reported as provisional. Allele frequencies are reported for gnomAD, the Turkish national variant database, and the in-house database in Table 1 and Table 2; these are presented for descriptive frequency context only, as no control cohort was available for statistical comparison. Segregation analysis in family members and loss of heterozygosity analysis in tumour tissue were not performed for any of the variants reported below.

### 3.3. Group 1 Genes: MCPH1 and Related Genes

Variants in Group 1 genes were identified in 11 of 185 patients (5.9%). The variants and clinical details of these patients are summarised in Table 1.

#### 3.3.1. MCPH1

Six patients carried heterozygous *MCPH1* variants. One variant met Likely Pathogenic criteria: a two-base-pair frameshift deletion, c.1869_1870del (p.Cys624Ter), identified in CASE 1, a 49-year-old female with invasive ductal carcinoma of the luminal B subtype (ER+/PR+/HER2+). This variant was reported in gnomAD at an allele frequency of 0.0000335 and in the Turkish national variant database at 0.000190. This classification was supported by the ACMG/AMP evidence codes PVS1 (null variant in a gene for which loss of function is an established disease mechanism), PM2 (absent/rare in population databases), and PP5 (previously reported as pathogenic/likely pathogenic by a reputable source).

Five additional *MCPH1* variants were classified as VUS. The c.2254C>G variant (p.Arg752Gly; CASE 2) had the highest in-house allele frequency among the *MCPH1* variants identified (0.001740) and was detected in a 49-year-old patient with invasive ductal carcinoma. The c.2351G>T variant (p.Gly784Val; CASE 3) is located within the BRCT2 domain and was absent from gnomAD; this patient also carried a concurrent *ESR1* VUS (c.884C>T, p.Pro295Leu, hormone receptor domain). The c.1361G>A variant (p.Ser454Asn; CASE 4), located within the Microcephalin domain, was identified in a 71-year-old female with invasive lobular carcinoma. The c.1517C>T variant (p.Ala506Val; CASE 5) was reported in gnomAD at an allele frequency of 0.00000186. The c.829T>C variant (p.Ser277Pro; CASE 6) is also located within the Microcephalin domain and was absent from both gnomAD and the national database; it was identified in a 37-year-old female.

#### 3.3.2. MDC1

Two patients carried heterozygous *MDC1* VUS. CASE 7 harboured c.394C>T (p.Arg132Cys) and was a 50-year-old female with breast cancer. CASE 8 carried c.4660C>T (p.Arg1554Trp) and presented with triple-negative breast cancer (ER−/PR−/HER2−) at age 38 with a Ki-67 proliferation index of 70%.

#### 3.3.3. TP53BP1

One patient (CASE 9) carried a heterozygous *TP53BP1* in-frame deletion, c.1362_1367del (p.Ile455_Pro456del). This variant was reported at an allele frequency of 0.008803 in gnomAD and 0.007000 in the national database. The patient was a 56-year-old female with breast cancer.

#### 3.3.4. TOPBP1

CASE 10 harboured a heterozygous *TOPBP1* missense VUS, c.1723A>G (p.Ile575Val). This variant was absent from the Turkish national database and was reported in gnomAD at an allele frequency of 0.0000167. The patient was a 34-year-old female with breast cancer.

#### 3.3.5. RAD52

One patient (CASE 11) carried a heterozygous *RAD52* nonsense variant, c.1037C>A (p.Ser346Ter). This variant had the highest population allele frequency among those detected in this study (gnomAD 0.01296; national database 0.029000) and was observed at an in-house frequency of 0.027060. The patient was a 50-year-old female with HER2-positive, hormone receptor-negative breast cancer (ER−/PR−/HER2+).

#### 3.3.6. UBE2N

No variants meeting the reporting criteria were identified in *UBE2N* across the 185 patients analysed.

### 3.4. Group 2 Genes: Candidate Genes with Low-Level Evidence

Variants in Group 2 candidate genes were detected in 9 of 185 patients (4.9%), with one additional concurrent *ESR1* variant identified in a patient counted in Group 1 (CASE 3). The variants and clinical details for this group are presented in Table 2.

#### 3.4.1. ATRIP

*ATRIP* was the gene in which variants were most frequently identified among the Group 2 candidates, with variants detected in four patients, all classified as VUS. CASE 12 carried c.32G>C (p.Arg11Thr) in a heterozygous state and presented with triple-negative breast cancer at age 41. CASE 13 carried c.2272C>T (p.Leu758Phe) in a homozygous state; this was the only homozygous variant detected in the study cohort, and the homozygous genotype was not recorded in either the Turkish national database or gnomAD. This patient was a 33-year-old female with ER+/PR+/HER2− breast cancer. CASE 14 (c.437G>A, p.Arg146Gln) was a 44-year-old female whose variant was absent from the national database. CASE 15 harboured a splice-region variant, c.1883-4del, and was a 55-year-old female with luminal A subtype breast cancer (ER+/PR+/HER2−). RNA-level analysis to assess the transcriptional consequence of this splice-region variant was not performed.

#### 3.4.2. ESR1

Two *ESR1* VUS were identified across the cohort. CASE 3, described above in relation to an *MCPH1* BRCT2-domain VUS (c.2351G>T), concurrently carried an *ESR1* missense variant, c.884C>T (p.Pro295Leu), located within the hormone receptor domain. CASE 16 carried an intronic *ESR1* variant, c.1097-12G>C, which was absent from the national database; this patient was a 67-year-old female with ER+/PR−/HER2+ breast cancer. As above, RNA-level analysis was not performed.

#### 3.4.3. PPM1D

CASE 17 carried a heterozygous *PPM1D* missense VUS, c.1113A>G (p.Ile371Met), located within the PP2C phosphatase domain. This variant was reported in gnomAD at an allele frequency of 0.00000186 and was absent from the national database. The patient was a 57-year-old female with breast cancer.

#### 3.4.4. RAD54L

*RAD54L* variants were identified in three patients. CASE 18 carried c.1759C>T (p.Arg587Trp), located within the HDA2-3 and Helicase_C domains, and was a 62-year-old female with invasive lobular carcinoma; this variant was reported in gnomAD at an allele frequency of 0.002294. CASE 19 carried c.923C>T (p.Thr308Ile), located within the ResIII, DEAD, and SNF2_N domains, and was the only male breast cancer patient among the variant-positive cases; this variant was reported in gnomAD at an allele frequency of 0.00000124. CASE 20 was identified as carrying a CNV comprising a duplication of exon 12 within the SNF2_N domain; this copy number finding was called from targeted panel read-depth data and has not been confirmed by an independent method, and is therefore reported as provisional.

#### 3.4.5. RRAS2 and XRCC2

No variants meeting the reporting criteria were identified in *RRAS2* or *XRCC2* in any of the 185 patients analysed.

## 4. Discussion

The present study investigated germline variants in *MCPH1* and functionally related genes, as well as selected low-evidence candidate genes, in 185 breast cancer patients whose first-tier evaluation with a 42-gene hereditary cancer panel and *BRCA1/2*/*PALB2* MLPA testing had been uninformative. Rare variants meeting reporting criteria were identified in 20 of 185 patients (10.8%), comprising one LP variant, nineteen VUS, and one provisional CNV. As no ethnically matched control cohort was available, these findings should be interpreted as descriptive rather than as evidence of gene-level enrichment or association with breast cancer susceptibility. This detection rate is broadly comparable to that reported in other second-tier or extended panel studies in *BRCA1/2*-negative populations. In a Turkish cohort from a comparable Aegean region centre, Solmaz et al. reported pathogenic or likely pathogenic variants in 11.1% of *BRCA1/2*-negative high-risk breast cancer patients tested with an extended multigene panel [16], and a large German study across approximately 7000 HBOC patients demonstrated that broadening genetic evaluation beyond *BRCA1/2* adds clinically meaningful diagnostic value [2]. It should be emphasised, however, that these comparisons are not directly equivalent: the figures cited from those studies refer to pathogenic and likely pathogenic findings in established genes, whereas the present detection rate is composed almost entirely of variants of uncertain significance in candidate genes with limited or unestablished disease association.

The predominance of VUS in the present cohort is consistent with the wider literature on candidate-gene and exome-based studies in *BRCA*-negative patients. Comparable investigations have reported that a substantial proportion of prioritised variants are VUS, and large case–control studies in populations underrepresented in reference databases have shown that pathogenic-variant identification and classification confidence improve substantially when ancestry-matched control data are available, addressing the classification uncertainty that the scarcity of such data otherwise produces in clinical testing [17]. The Turkish population is similarly underrepresented in international reference datasets, which plausibly contributes both to the high VUS proportion observed here and to the absence of several detected variants from gnomAD. Notably, studies that have been able to move beyond descriptive reporting in this setting have done so by incorporating ancestry-matched control cohorts and formal case–control burden testing [6,17]; the absence of such a control group is the principal methodological difference between those studies and the present work, and defines the necessary next step for the candidate genes examined here.

The most notable finding of the present study was an *MCPH1* frameshift deletion, c.1869_1870del, in CASE 1, a 49-year-old patient with luminal B invasive ductal carcinoma. Frameshift variants of this nature are expected to cause premature termination and loss of protein function, which is consistent with the tumour suppressor role attributed to *MCPH1* in the DNA damage response and homologous recombination [18,19]. Reduced *MCPH1* expression has been documented across multiple cancer types, and experimental data have implicated *MCPH1* loss in genomic instability in breast epithelial cells [19,20]. Five further *MCPH1* VUS were identified, two of which—c.2351G>T and c.829T>C—were absent from both gnomAD and the national database. While the c.2351G>T variant lies within the BRCT2 domain, which mediates protein interactions in DNA damage signalling [21], domain location alone does not establish functional consequence, and these variants remain of uncertain significance in the absence of functional or segregation data. The identification of six *MCPH1* variants among 185 patients (3.2%), one meeting LP criteria, is therefore best regarded as a preliminary observation warranting evaluation in larger, controlled cohorts.

Among the remaining Group 1 genes, two *MDC1* VUS, one *TP53BP1* VUS, one *TOPBP1* VUS, and one *RAD52* nonsense variant were identified. *MDC1* encodes a scaffolding protein that amplifies DNA damage response signalling at double-strand breaks [22] and has been shown to co-regulate centrosome integrity with *BRIT1* in breast cancer cells [23]. The *MDC1* variant in CASE 8 was identified in a patient with triple-negative breast cancer diagnosed at age 38 with a Ki-67 index of 70%; while this clinical profile is of interest, no inference regarding the pathogenicity of the variant can be drawn from a single case. The *TP53BP1* in-frame deletion in CASE 9 carries a gnomAD allele frequency of 0.008803, which is incompatible with a rare high-penetrance susceptibility allele and argues against clinical relevance in this context. Conversely, the *TOPBP1* variant in CASE 10 was rare and absent from the national database, and was identified in a 34-year-old patient; *TOPBP1* functions as a scaffold for *ATR* activation during replication stress [24], but the significance of this single observation cannot be determined without further data. The *RAD52* nonsense variant in CASE 11 corresponds to the previously described S346X allele, which Adamson et al. reported to be associated with reduced breast cancer risk in *BRCA2* mutation carriers [25]. Its population frequency in this study (gnomAD 0.01296; national database 0.029) is consistent with a common polymorphism rather than a susceptibility allele, and its detection here should not be interpreted as evidence of pathogenic contribution.

*ATRIP* was the gene in which variants were most frequently identified among the Group 2 candidates, with four VUS detected. *ATRIP* encodes the obligate binding partner of *ATR* kinase, and the *ATR*–*ATRIP* complex is required for checkpoint signalling under replication stress [26,27]. The homozygous variant c.2272C>T in CASE 13, a 33-year-old patient, was the only homozygous variant in the cohort, and the homozygous genotype was not recorded in gnomAD or the national database. Biallelic variants in *ATR* itself have been associated with Seckel syndrome, whereas the phenotypic consequences of biallelic *ATRIP* variation in humans remain undefined. This observation is therefore reported as a candidate for future segregation and functional analysis rather than as evidence of a pathogenic genotype; the absence of parental and family samples in the present study precludes any assessment of inheritance or co-segregation.

Three *RAD54L* variants were identified. *RAD54L* encodes an SWI2/SNF2-family chromatin remodelling enzyme that cooperates with RAD51 during homologous recombination. CASE 19 carried a rare missense variant affecting conserved helicase domains and was the only male breast cancer patient among the variant-positive cases. Male breast cancer accounts for less than 1% of breast cancers and is enriched for hereditary susceptibility, which makes this observation of interest, although a single case cannot support an association. CASE 20 carried a duplication of exon 12; as this copy number finding was called from targeted panel read-depth data without orthogonal confirmation, it remains provisional, and copy number variants in DNA repair genes generally require dedicated confirmation before any clinical interpretation. These observations are consistent with a recent exome sequencing study in Tunisian patients with inflammatory breast cancer, which identified a rare germline *RAD54L* missense variant; in that study, copy number variants were separately detected in other DNA repair genes (*ABRAXAS1*, *XRCC2*, and Fanconi anaemia pathway genes), not in *RAD54L* itself [28].

Two *ESR1* VUS and one *PPM1D* VUS were identified. *ESR1* mutations in clinical oncology are predominantly somatic, arising in the metastatic setting as a mechanism of endocrine therapy resistance [29,30]; whether germline *ESR1* alterations confer breast cancer susceptibility independently of this established somatic role is unresolved, and the two variants identified here do not meet criteria for pathogenicity. *PPM1D* attenuates the *ATM*/*ATR*-mediated damage response by dephosphorylating p53 and CHK2, and truncating gain-of-function variants in exon 6 have been reported in blood DNA of cancer patients [31,32]; the variant identified in CASE 17 lies within the enzymatic domain rather than exon 6, and its functional consequence is unknown. No variants meeting reporting criteria were identified in *UBE2N*, *RRAS2*, or *XRCC2*. While absence of variants does not exclude a role for these genes, the negative findings are consistent with the limited and inconsistent evidence currently available, particularly for *XRCC2*, for which polymorphism studies across solid tumours have yielded inconclusive results regarding breast cancer risk [33].

Prioritisation for future functional and segregation studies. Among the variants reported here, four are considered the highest priority for dedicated follow-up. The *MCPH1* frameshift deletion (c.1869_1870del, CASE 1) is the top priority given its likely pathogenic classification, and warrants family segregation analysis and, where feasible, RNA- or protein-level functional confirmation. The homozygous *ATRIP* variant (c.2272C>T, CASE 13) merits parental testing and segregation analysis to establish inheritance, given the undefined phenotypic consequence of biallelic *ATRIP* variation in humans. The two splice-region variants (*ATRIP* c.1883-4del and *ESR1* c.1097-12G>C) should be prioritised for RNA-level analysis to determine their transcriptional consequence. Finally, the *RAD54L* exon 12 duplication (CASE 20) requires orthogonal confirmation by MLPA or array-based analysis before any further evaluation, as its provisional status precludes prioritisation for functional study at this stage. Two further variants merit note as secondary candidates: the *RAD54L* missense variant (c.923C>T, CASE 19), given its extreme rarity, location within conserved helicase domains, and detection in the only male breast cancer patient in the cohort—a phenotype recognised to be enriched for hereditary susceptibility—and the two *MCPH1* variants absent from both gnomAD and the national database (c.2351G>T within the BRCT2 domain, CASE 3; c.829T>C within the Microcephalin domain, CASE 6), given their novelty and location within annotated functional domains of the gene most central to this study. Conversely, the *TP53BP1* in-frame deletion (CASE 9) and the *RAD52* nonsense variant (CASE 11) are not prioritised for further functional or segregation study, as their comparatively high population allele frequencies (gnomAD 0.008803 and 0.01296, respectively) are inconsistent with rare, highly penetrant alleles.

### Limitations

Several limitations constrain the interpretation of these findings. First, and most importantly, no ethnically matched control cohort was available, and consequently no case–control burden analysis, enrichment testing, or estimation of association measures (odds ratios, confidence intervals, *p*-values) could be performed. The study is therefore descriptive, and no association between the candidate genes examined and hereditary breast cancer susceptibility is established by these data. Second, nineteen of the twenty-one variants reported are of uncertain significance; definitive classification requires functional characterisation, family segregation analysis, and accumulation of independent case-level evidence, none of which was available here. Third, co-segregation analysis in affected relatives and loss of heterozygosity analysis in tumour tissue—the principal lines of evidence used to support causality in hereditary cancer—were not undertaken. Fourth, RNA-level analysis was not performed, so the transcriptional consequences of the splice-region variants identified (*ATRIP* c.1883-4del and *ESR1* c.1097-12G>C) remain unknown. Fifth, the sequencing strategy targeted coding regions and exon–intron boundaries (±10 bp) only; large structural rearrangements such as balanced translocations and inversions, and deep intronic variants of potential functional significance, were not analysed, and pathogenic variants outside the sequenced intervals may have been missed. Sixth, the copy number variant reported in CASE 20 was derived from read-depth analysis of targeted panel data and was not confirmed by an independent method; Sanger confirmation was applicable only to the 20 sequence variants. Seventh, although age at diagnosis, family history, and bilateral disease status were available for the full cohort (Section 3.1), molecular subtype could be assigned for only 94 of 185 patients (50.8%), chiefly owing to missing Ki-67 values, and other prognostic variables (tumour stage, grade, treatment response) were not systematically recorded; this precludes a comprehensive genotype–phenotype comparison against variant-negative patients. Finally, the cohort size of 185 patients, while adequate for descriptive variant discovery, provides limited power for genotype–phenotype analysis within individual gene subgroups and does not permit estimation of penetrance.

## 5. Conclusions

In this study, a second-tier analysis of *MCPH1*, functionally related DNA damage response genes, and selected low-evidence candidate genes was performed in 185 breast cancer patients who remained genetically unresolved after standard hereditary cancer panel testing and *BRCA1/2*/*PALB2* MLPA analysis. Rare variants meeting reporting criteria were identified in 20 patients (10.8%): one likely pathogenic *MCPH1* frameshift deletion, nineteen variants of uncertain significance, and one unconfirmed copy number variant. The candidate gene selection strategy, based on GenesLikeMe similarity scoring and the Genomics England PanelApp gene list, offers a transparent and reproducible basis for assembling a biologically coherent gene set for exploratory analysis.

As detailed in the Limitations, the descriptive design and the absence of an ethnically matched control cohort mean that these data do not establish an association between the genes examined and hereditary breast cancer susceptibility. The genes analysed here therefore remain research candidates rather than genes suitable for clinical diagnostic panels; definitive assessment will require case–control studies with ancestry-matched controls, formal burden testing, family segregation analysis, and functional characterisation of individual variants.

The predominance of variants of uncertain significance also carries clinical implications. VUS are not clinically actionable, must not be used for predictive testing in unaffected relatives, and require clear counselling regarding their uncertain status; without this, they may cause unnecessary anxiety or prompt inappropriate management decisions. We nevertheless consider their systematic documentation and follow-up to be clinically justified. Published series indicate that a meaningful proportion of VUS in cancer predisposition genes are subsequently upgraded to pathogenic or likely pathogenic as evidence accumulates [34,35,36], and reassessment of clinically relevant VUS at approximately two-year intervals is recommended [35]. This is particularly relevant to the Turkish population: in a cohort of Turkish breast and colorectal cancer patients, re-evaluation of 415 previously reported VUS after five years led to reclassification of 98 (23.6%) as benign or likely benign and 16 (3.9%) as pathogenic or likely pathogenic, increasing the positive test rate among breast cancer patients by 3.7% [6]. At our centre, VUS are reported within this framework and carriers are enrolled in periodic review, so that reclassification can be acted upon when it occurs. Where candidate genes are analysed for research purposes, we consider it appropriate that reporting practice be aligned with the evidence level of the gene, and that variants in genes below Moderate ClinGen classification be presented as research findings rather than clinical results. The present findings are offered in this spirit: as preliminary observations intended to inform the design of future studies.

## Figures and Tables

**Table 1:** Variants identified in *MCPH1* and related genes (Group 1).

Case No.	Gene	Variant (cDNA)	Protein	Domain	Classification	Zygosity	gnomAD AF	National AF	In-House AF	Clinical Data
1	*MCPH1*	c.1869_1870del	p.Cys624Ter	–	LP	Het	0.0000335	0.000190	0.000320	F/49/IDC/ER+PR+HER2+
2	*MCPH1*	c.2254C>G	p.Arg752Gly	–	VUS	Het	0.0000607	0.001300	0.001740	F/49/IDC
3	*MCPH1*	c.2351G>T	p.Gly784Val	BRCT2	VUS	Het	Novel	0.000490	0.000750	F/48/BC
3 *	*ESR1*	c.884C>T	p.Pro295Leu	Hormone receptor	VUS	Het	0.0000155	0.000190	0.000300	Concurrent (same patient)
4	*MCPH1*	c.1361G>A	p.Ser454Asn	Microcephalin	VUS	Het	0.0000105	0/0	0.000190	F/71/ILC/ER+PR+HER2−
5	*MCPH1*	c.1517C>T	p.Ala506Val	–	VUS	Het	0.00000186	0/0	0.000210	F/60/BC
6	*MCPH1*	c.829T>C	p.Ser277Pro	Microcephalin	VUS	Het	Novel	0/0	0.000190	F/37/BC
7	*MDC1*	c.394C>T	p.Arg132Cys	–	VUS	Het	0.0000322	0.000490	0.000300	F/50/BC
8	*MDC1*	c.4660C>T	p.Arg1554Trp	–	VUS	Het	0.000361	0.000190	0.000590	F/38/TNBC/Ki67:70%
9	*TP53BP1*	c.1362_1367del	p.Ile455_Pro456del	–	VUS	Het	0.008803	0.007000	0.007160	F/56/BC
10	*TOPBP1*	c.1723A>G	p.Ile575Val	–	VUS	Het	0.0000167	0/0	0.000450	F/34/BC
11	*RAD52*	c.1037C>A	p.Ser346Ter	–	VUS	Het	0.01296	0.029000	0.027060	F/50/ER−PR−HER2+

AF: allele frequency; BC: breast cancer; F: female; Het: heterozygous; Hom: homozygous; IDC: invasive ductal carcinoma; ILC: invasive lobular carcinoma; LP: likely pathogenic; M: male; Novel: not previously reported in the referenced population database; TNBC: triple-negative breast cancer; VUS: variant of uncertain significance. *: concurrent variant in the same patient as CASE 3.

**Table 2:** Variants identified in candidate genes (Group 2).

Case No.	Gene	Variant (cDNA)	Protein	Domain	Classification	Zygosity	gnomAD AF	National AF	In-house AF	Clinical Data
12	*ATRIP*	c.32G>C	p.Arg11Thr	–	VUS	Het	0.0000107	0.000490	0.000910	F/41/TNBC
13	*ATRIP*	c.2272C>T	p.Leu758Phe	–	VUS	Hom	Novel hom	Novel hom	0.000900	F/33/ER+PR+HER2−
14	*ATRIP*	c.437G>A	p.Arg146Gln	–	VUS	Het	0.00000808	0/0	0.000450	F/44/BC
15	*ATRIP*	c.1883-4del	–	–	VUS	Het	0.0000124	0.000190	0.000450	F/55/ER+PR+HER2−
16	*ESR1*	c.1097-12G>C	–	–	VUS	Het	0.00000372	0/0	0.000600	F/67/ER+PR−HER2+
17	*PPM1D*	c.1113A>G	p.Ile371Met	PP2C	VUS	Het	0.00000186	0/0	0.000200	F/57/BC
18	*RAD54L*	c.1759C>T	p.Arg587Trp	HDA2-3/Helicase_C	VUS	Het	0.002294	0.000290	0.001210	F/62/ILC
19	*RAD54L*	c.923C>T	p.Thr308Ile	ResIII/DEAD/SNF2_N	VUS	Het	0.00000124	0.000580	0.000530	M/69/BC
20	*RAD54L*	CNV Exon 12 dup	–	SNF2_N	CNV	–	–	–	–	F/58/BC

AF: allele frequency; BC: breast cancer; CNV: copy number variant; F: female; Het: heterozygous; Hom: homozygous; ILC: invasive lobular carcinoma; Novel hom: homozygous state not previously observed in national or gnomAD databases; M: male; TNBC: triple-negative breast cancer; VUS: variant of uncertain significance.

## Data Availability

The original contributions presented in this study are included in the article/Supplementary Material. Further inquiries can be directed to the corresponding author.

## Supplementary Materials

The following supporting information can be downloaded at: https://www.mdpi.com/article/10.3390/diagnostics16182967/s1, Table S1: List of the 42 genes included in the custom hereditary cancer panel with their corresponding Ensembl transcript (ENST) identifiers.

## Abbreviations

The following abbreviations are used in this manuscript:

ACMG	American College of Medical Genetics and Genomics
AF	Allele Frequency
*ATM*	Ataxia Telangiectasia Mutated
*ATR*	Ataxia Telangiectasia and Rad3-related
B	Benign
BC	Breast Cancer
BRCT	*BRCA1* C-Terminal domain
CHK1	Checkpoint Kinase 1
CHK2	Checkpoint Kinase 2
CNV	Copy Number Variant
DNA	Deoxyribonucleic Acid
ER	Estrogen Receptor
F	Female
GRCh38	Genome Reference Consortium Human Build 38
HBOC	Hereditary Breast and Ovarian Cancer
HER2	Human Epidermal Growth Factor Receptor 2
Het	Heterozygous
Hom	Homozygous
IDC	Invasive Ductal Carcinoma
IGV	Integrative Genomics Viewer
ILC	Invasive Lobular Carcinoma
LB	Likely Benign
LP	Likely Pathogenic
M	Male
*MCPH1*	Microcephalin 1 (*BRIT1*)
MiDAS	Mitotic DNA Synthesis
MLPA	Multiplex Ligation-dependent Probe Amplification
mRNA	Messenger RNA
MRN	MRE11-RAD50-NBS1 complex
NCCN	National Comprehensive Cancer Network
NGS	Next-Generation Sequencing
P	Pathogenic
PARP	Poly (ADP-Ribose) Polymerase
PLK1	Polo-like Kinase 1
PP2C	Protein Phosphatase 2C
PR	Progesterone Receptor
TNBC	Triple-Negative Breast Cancer
VUS	Variant of Uncertain Significance
